# A multi-institutional feasibility study of S-1/oxaliplatin plus bevacizumab in patients with advanced/metastatic colorectal cancer: the HiSCO-02 prospective phase II study

**DOI:** 10.1186/s40064-016-3491-8

**Published:** 2016-10-18

**Authors:** Manabu Shimomura, Katsunori Shinozaki, Takao Hinoi, Masanori Yoshimitsu, Manabu Kurayoshi, Daisuke Sumitani, Yasuyo Ishizaki, Takafumi Oshiro, Shinya Kodama, Yosuke Shimizu, Michinori Arita, Masakazu Tokunaga, Makoto Yoshida, Junko Tanaka, Hideki Ohdan, Hiroshima Surgical study group of Clinical Oncology (HiSCO)

**Affiliations:** 1 Department of Gastroenterological and Transplant Surgery, Applied Life Sciences, Institute of Biomedical and Health Sciences, Hiroshima University, Hiroshima, Japan; 2 Division of Clinical Oncology, Hiroshima Prefectural Hospital, 1-5-54, Ujina-Kanda, Minami-ku, Hiroshima, Japan; 3 Department of Surgery, Hiroshima City Asa Hospital, Hiroshima, Japan; 4 Department of Surgery, National Hospital Organization Higashihiroshima Medical Center, Higashihiroshima, Japan; 5 Department of Surgery, Onomichi General Hospital, Onomichi, Japan; 6 Department of Surgery, Chugoku Rosai Hospital, Kure, Japan; 7 Department of Surgery, Hiroshima General Hospital of West Japan Railway Company, Hiroshima, Japan; 8 Department of Surgery, Yoshida General Hospital, Akitakata, Japan; 9 Department of Surgery, National Hospital Organization Kure Medical Center, Kure, Japan; 10 Department of Surgery, Chuden Hospital, Hiroshima, Japan; 11 Department of Surgery, National Hospital Organization Hiroshima-nishi Medical Center, Ohtake, Japan; 12 Department of Surgery, Kure City Medical Association Hospital, Kure, Japan; 13 Department of Epidemiology, Infectious Disease Control and Prevention, Institute of Biomedical and Health Sciences, Hiroshima University, Hiroshima, Japan

**Keywords:** Metastatic colorectal cancer, Chemotherapy, S-1, Prospective phase II study

## Abstract

**Purpose:**

FOLFOX is a standard combination chemotherapy regimen for metastatic colorectal cancer (CRC). 5-Fluorouracil (5-FU) is infused continuously through a pump for 46 h; therefore, replacement of infused 5-FU with oral S-1 would be more convenient for patients. We investigated the efficacy and safety of S-1/oxaliplatin (SOX) plus bevacizumab regimen in a community setting.

**Methods:**

We conducted a phase II clinical study in Hiroshima, Japan. We enrolled individuals aged 20–80 years who had metastatic CRC, an Eastern Cooperative Oncology Group performance status of 0 or 1, assessable lesions, and not received previous chemotherapy. Eligible patients were administered SOX plus bevacizumab (S-1 80 mg/m^2^/day, day 1–14 orally; and oxaliplatin 130 mg/m^2^ day 1 i.v., bevacizumab 7.5 mg/kg, day 1 i.v. q3w). The primary endpoint was response rate (RR), and the secondary endpoints were progression-free survival (PFS), overall survival (OS), and safety.

**Results:**

Between May 2011 and January 2014, 55 patients (mean age 64 years) were enrolled at 12 institutions. Median follow up duration was 20.2 months (range 1.3–47.1 months). RR was 47.1 % [95 % confidence interval (CI) 33.7–60.6 %]. Median PFS and OS was 9.2 months (95 % CI 7.6–10.8) and 22.5 months (95 % CI 19.4–25.9), respectively. Major adverse events (grade 3/4) were neutropenia (9.3 %), thrombocytopenia (5.6 %), anorexia (18.5 %), and sensory neuropathy (16.7 %).

**Conclusion:**

These data suggested that SOX plus bevacizumab is effective and capable of being managed in metastatic CRC patients in our community clinical practice.

## Introduction

The combination chemotherapies, FOLFOX (5-fluorouracil [5-FU], leucovorin, and oxaliplatin) and FOLFIRI (5-FU, leucovorin, and irinotecan), have been the most common first-line metastatic colorectal cancer (CRC). Targeted agents that enhance the effect of chemotherapy have been discovered, including bevacizumab (a humanized monoclonal antibody that targets vascular endothelial growth factor, a central regulator of angiogenesis), cetuximab and panitumumab (monoclonal antibodies directed against the epidermal growth factor receptor) (Hurwitz et al. 2004; Van Cutsem et al. 2009).

FOLFOX with bevacizumab is widely used in clinical practice as the first line treatment for metastatic CRC (Goldberg et al. 2004). However, this regimen is inconvenient owing to its requirement for continuous infusion via vascular access. To overcome this drawback, oral fluoropyrimidines, such as capecitabine, have been used as a substitute for infused leucovorin and fluorouracil. Recent data have demonstrated that capecitabine and oxaliplatin plus bevacizumab is non-inferior to FOLFOX plus bevacizumab regimen considering progression-free survival (PFS) of patients with metastatic CRC (Cassidy et al. 2008; Saltz et al. 2008).

Another oral fluorouracil, S-1, is a chemotherapy agent that consists of tegafur (a pro-drug of 5-FU) and two agents, gimeracil and oteracil, which decrease the rate of degradation of 5-FU anti-metabolite. S-1 could have advantages over capecitabine in terms of reducing the frequency of toxicities such as hand-foot syndrome (HFS), and several trials have shown the feasibility and efficacy of S-1/oxaliplatin (SOX) for metastatic CRC (Yamada et al. 2008; Zang et al. 2009). However, there is still a lack of sufficient evidence of efficacy and safety for this new treatment regimen to become a standard choice in clinical practice.

Accordingly, we conducted a multicenter clinical phase II trial across 12 institutions in Hiroshima, Japan (the Hiroshima Surgical study group of Clinical Oncology; HiSCO). We aimed to investigate the efficacy and safety of SOX plus bevacizumab, a promising alternative treatment for metastatic CRC.

## Patients and methods

### Patient selection

We undertook an open-label, non-randomized, multicenter clinical phase II trial in 12 institutions in Hiroshima, Japan. We enrolled individuals who met the following eligibility criteria: (1) histologically proven colorectal adenocarcinoma; (2) unresectable advanced/metastatic CRC; (3) aged 20–80 years; (4) Eastern Cooperative Oncology Group performance status (PS) of 0 or 1; (5) presence of assessable lesions as confirmed on computed tomography or magnetic resonance imaging; (6) no previous chemotherapy or radiotherapy; (7) could take drugs orally; (8) adequate hematological, renal, and hepatic functions, as defined by a leucocyte count of 3–12 × 10^9^/L; neutrophil count of at least 2.0 × 10^9^/L; platelet count of at least 100 × 10^9^/L; hemoglobin level of at least 9.0 g/dL; total serum bilirubin concentration of no more than 1.5 mg/dL; serum aspartate aminotransferase/serum alanine aminotransferase concentration of no more than 100 U/L; serum creatinine concentration of no more than 1.2 mg/dL; creatinine clearance >60 mL/min; urinary protein score of no more than 1+; and an international normalized ratio of no more than 1.5 and (9) estimated life expectancy of >3 months.

We excluded individuals if they had a history of serious allergies to any medications, active infections, serious concurrent disease, substantially impaired cardiac function, gastrointestinal ulcers or bleeding, sensory neuropathy, serious diarrhea, ascites or pleural effusion needing medication, brain metastases, a history of gastrointestinal perforation within the 6 months before enrollment, a history of thromboembolism or interstitial pneumonia, a history of surgery within the 28 days before enrollment, a blood coagulation disorder, were on anticoagulation medication, a history of hemoptysis, or had a primary lesion associated with a severe stricture that precluded passage of an endoscope. We also excluded individuals if they had previously or were presently receiving oxaliplatin-based regimens as adjuvant chemotherapy.

The current study was conducted in accordance with the declaration of Helsinki. All patients provided written informed consent after having been informed about the purpose and investigational nature of the study. The institutional review boards or ethics committees of each of the participating centers reviewed and approved the protocol. This study was registered in the UMIN Clinical Trial Registry as UMIN000004976.

### Treatment

On day 1 of each 3-week cycle, patients assigned to receive SOX plus bevacizumab received a 7.5 mg/kg intravenous infusion of bevacizumab (for 30–90 min), followed by an intravenous infusion of 130 mg/m^2^ oxaliplatin (for 2 h). S-1 was taken orally twice daily from after dinner on day 1 to after breakfast on day 15, followed by a 7-day break. The dose of S-1 was assigned according to body surface area: patients with a body surface area of less than 1.25 m^2^ received 80 mg/day; those with a body surface area between 1.25 m^2^ and less than 1.5 m^2^ received 100 mg/day; and those with a body surface area of at least 1.5 m^2^ received 120 mg/day. Cycles were repeated for each patient until the criteria for withdrawal of the study treatment were met.

In view of the neurological toxicity of oxaliplatin, treatment could be skipped when patients had received at least 600 mg/m^2^ overall, even when no grade 3 toxic effects were recorded. If patients had grade 2 or higher proteinuria or grade 2 or higher bleeding before the scheduled starting day of each cycle, they received only SOX treatment; bevacizumab could be resumed in subsequent cycles if the treatment criteria were satisfied. The dose of cytotoxic drugs (oxaliplatin and S-1) was reduced by one level if the neutrophil count was less than 0.5 × 10^9^/L at any time during a cycle, the neutrophil count was less than 1.0 × 10^9^/L on the first day of a cycle, grade 3 or higher febrile neutropenia developed, or the platelet count was less than 50 × 10^9^/L. In the event of grade 3 or higher diarrhea, the dose of S-1 was reduced by one level. If the platelet count was between 50 × 10^9^ and 75 × 10^9^/L at any time during a cycle, or between 75 × 10^9^ and 100 × 10^9^/L on the first day of a cycle, the oxaliplatin dose was reduced by one level. S-1 was withheld when the neutrophil count was less than 1 × 10^9^/L; the platelet count was less than 75 × 10^9^/L; the serum creatinine concentration was more than 1.5 mg/dL; suspected infection was diagnosed due to a fever of at least 38 °C; or diarrhea, mucositis, or stomatitis of grade 2 or higher developed. S-1 was subsequently reinitiated when the neutrophil count was at least 1 × 10^9^/L; the platelet count was at least 75 × 10^9^/L; the serum creatinine concentration was less than 1.5 mg/dL; no fever of 38 °C or higher suggesting infection was evident; and diarrhea, mucositis, and stomatitis were no higher than grade 1.

### Endpoints

The primary endpoint of this study was response rate (RR), and the secondary endpoints were PFS, overall survival (OS), and safety.

RR was calculated for patients who had measurable lesions using the Response Evaluation Criteria in Solid Tumor (RECIST; version 1.1) (Eisenhauer et al. 2009). RR and disease control rate (DCR) were analyzed for the patients with target lesions. After initiation of study treatment, target and non-target lesions were assessed every 8 weeks in the same way as at baseline, using the same imaging conditions.

PFS was defined as the interval from the date of enrollment to the date on which progressive disease was first confirmed or the date of death from any cause, whichever came first. OS was defined as the interval from the date of enrollment to the date of death from any cause or last follow-up. Adverse events were graded according to the Common Terminology Criteria for Adverse Events (CTCAE; version 4.0).

We also evaluated the proportion of patients achieving disease control (a complete or partial response or stable disease), the proportion of patients having a curative resection, the time to treatment failure (TTF, interval from the date of enrolment to the date of a PFS event or withdrawal from the study for any reason), and adverse events.

### Statistical analysis

All endpoints analyses except for the safety analysis were performed on the intent-to-treat set. The safety analysis included all treated patients who received at least one dose of the experimental drug. The required sample size was calculated to be at least 55 patients on the null hypothesis of a RR of 30 % versus the alternative hypothesis of a RR of 50 %, with a power of 80 %, and a 95 % significance level (one sided). Survival curves were estimated using the Kaplan–Meier method. All statistical analyses were performed using the IBM SPSS Statistic 20.0 software package.

## Results

### Patient characteristics

Between May 2011 and January 2014, 55 patients were enrolled in this study. Patient characteristics are listed in Table 1. The mean age was 64 years. Forty-nine patients had a PS of 0, and 6 had a PS of 1. Of the 55 patients, 37 underwent primary tumor resection (67.3 %), and 12 underwent adjuvant chemotherapy (21.8 %). The number of organs with metastatic lesions was one in 29 patients (52.7 %) and two or more in 25 patients (45.4 %), and 2 patients had no assessable lesions (3.6 %). The median follow-up duration was 20.2 months (range 1.3–47.1).

**Table 1:** Baseline patient characteristics (n = 55)

Parameter	Number of patients
*Sex*	
Male	36
Female	19
Age, years (range)	64 (21–79)
*Performance status*	
0	49
1	6
*Primary site*	
Colon	33
Rectosigmoid	3
Rectum	19
*Tumor differentiation*	
well	13
moderate	36
poor	2
others	3
unknown	1
*Adjuvant chemotherapy*	
Yes (%)	12 (21.8 %)
Uracil and tegafur plus leucovorin	5
Capecitabine	5
5-FU/LV	1
Uracil and tegafur	1
Primary tumor resection (%)	37 (67.3 %)
*Assessable lesion*	
No	2
Yes	53
*Metastatic sites*	
Liver	32
Lung	23
Lymph node metastases	13
Peritoneal dissemination	4
other metastases	11
*Number of metastatic sites*	
1	29
2	17
≥3	9
Liver limited disease (%)	14 (25.4 %)
CEA (median, range)	21.2 (2.3–8577.6)
CA19-9 (median, range)	38.8 (0.7–328,230)

*5-FU* 5-fluorouracil, *LV* leucovorin, *CEA* carcinoembryonic antigen, *CA19-9* carbohydrate antigen 19-9

### Efficacy

One patient did not meet the eligibility criteria because of the absence of recurrent disease. Treatment was permanently stopped before the first tumor response evaluation for 4 patients owing to withdrawal of consent by 1 and owing to adverse events in the other 3 (grade 2 deep vein thrombosis in 1 patient, grade 3 anorexia in 1 patient, grade 4 neutropenia in 1 patient). In total, 472 treatment cycles were administered with a median of 7.5 cycles (range 0–34) per patient. S-1 was administrated in 472 cycles, oxaliplatin was administrated in 392 cycles, and bevacizumab was administrated in 423 cycles.

**Fig. 1:**
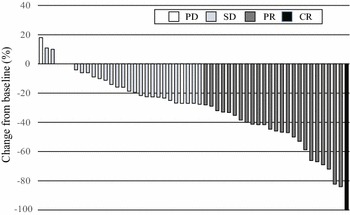
Waterfall plot analysis of the best overall response in the intention to treat set. *CR* complete response; *PR* partial response; *SD* stable disease; *PD* progressive disease

The tumor response data are listed in Table 2. RR was 47.1 % [95 % confidence interval (CI) 33.7–60.6 %], and therefore, the primary endpoint was achieved. DCR was 88.7 % (95 % CI 80.1–97.2 %), and median TTF was 6.3 months (95 % CI 4.0–8.5 months). RR and DCR were analyzed for the patients with assessable lesions. A curative R0 resection was performed in 4 patients (7.4 %). A waterfall plot of the best overall response is demonstrated in Fig. 1.

**Table 2:** Treatment outcomes

Outcomes	No. of patients (n = 53)^a^	95 % confidence interval
Response		
Complete response	1	
Partial response	24	
Stable disease	22	
Progressive disease	2	
Not evaluable	4	
Response rate (%)	25 (47.1 %)	33.7–60.6
Disease control rate (%)	47 (88.7 %)	80.1–97.2

^a^Response rate and disease control rate were analyzed for the patients with assessable lesion

**Fig. 2:**
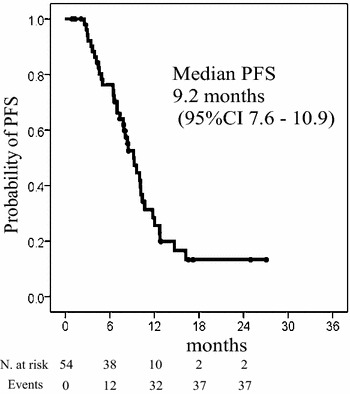
Kaplan-Meier curves for progression-free survival (PFS); the median PFS was 9.2 months (95 % confidence interval: 7.6–10.8) N. at risk, number at risk

**Fig. 3:**
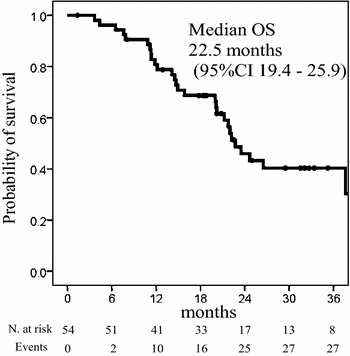
Kaplan-Meier curves for overall survival (OS); the median OS was 22.5 months (95 % confidence interval 19.4–25.9 months) N. at risk, number at risk

Median PFS was 9.2 months (95 % CI 7.5–10.9, Fig. 2), and median OS was 22.5 months (95 % CI 19.4–25.9 months, Fig. 3).

### Safety

The safety analysis included all treated patients who received at least one dose of the experimental drug (n = 54). The adverse events are listed in Table 3. Major hematological adverse events (grade 3/4) were neutropenia (9.3 %), thrombocytopenia (5.6 %), and leucopenia (5.6 %). Major non-hematological adverse events (grade 3/4) were hypertension (22.2 %), anorexia (18.5 %), and sensory neuropathy (16.7 %). The toxic effects were capable of being managed; however, attention to the occurrence of anorexia was needed.

**Table 3:** Relative dose intensity

Drugs	Cycles	Median (%)	Range (%)
S-1	472	84	23–100
Oxaliplatin	392	88	52–100
Bevacizumab	423	90	31–100

The median relative dose intensities (RDIs; ratio of dose received to dose planned) are listed in Table 4. The median RDI of oxaliplatin, S-1, and bevacizumab was 84, 88, and 90 %, respectively.

**Table 4:** Safety analysis (n = 54)

	Any grade	>Grade3
	n (%)	n (%)
*Haematological*		
Leucopenia	30 (55.6)	3 (5.6)
Neutropenia	30 (55.6)	5 (9.3)
Thrombocytopenia	35 (61.1)	3 (5.6)
Increased aspartate aminotransferase or alanine aminotransferase concentration	33 (61.1)	0 (0)
Increased creatinine concentration	8 (14.8)	0 (0)
Proteinurea	20 (37)	0 (0)
*Non-haematological*		
Mucositis or stomatitis	16 (29.6)	1 (1.9)
Anorexia	38 (70.4)	10 (18.5)
Nausea	16 (29.6)	3 (5.6)
Vomiting	10 (18.5)	0 (0)
Diarrhoea	16 (29.6)	0 (0)
Rash or desquamation	11 (20.4)	0 (0)
Hyperpigmentation	14 (25.9)	0 (0)
Fatigue	36 (68.7)	1 (1.9)
Sensory neuropathy	37 (68.5)	9 (16.7)
Hypertension	46 (85.2)	12 (22.2)
Alopecia	1 (1.9)	0 (0)
Hand-foot syndrome	8 (14.8)	0 (0)
Gastroinetestinal obstrucion	4 (7.4)	3 (5.6)
Gastrointestinal perforation	0 (0)	0 (0)
Fever	4 (7.4)	1 (1.9)
Thrombosis, thrombus, or embolism	2 (3.8)	1 (1.9)
Bleeding complication	5 (9.2)	1 (1.8)

## Discussion

We herein demonstrated that SOX plus bevacizumab is effective and capable of being managed in metastatic CRC patients in our community clinical practice.

The combination of chemotherapeutic regimens and the development of molecular targeted agents lead to improved survival in metastatic CRC. In order to develop new chemotherapeutic regimens, it is important to consider not only the survival benefit but also the maintenance of the quality of life of patients. S-1 is an effective derivative that combines tegafur with two modulators of 5-FU metabolism, gimeracil, a reversible inhibitor of dihydropyrimidine dehydrogenase, and oteracil in a molar ratio of 1:0.4:1. Gimeracil maintains a high fluorouracil concentration in the blood for a long time, and oteracil inhibits the conversion of 5-FU to active metabolites in the gastrointestinal tract, resulting in a reduction in gastrointestinal toxicity (Kato et al. 2001; Shirasaka 2009). S-1 was originally approved for the treatment of gastric cancer in Japan in 1999, and subsequently gained Japanese approval for CRC in 2003. More recently, there has been a shifting paradigm in cancer care towards oral chemotherapeutics. In the view of the convenience oral dosing offers to both patients and physicians, S-1 was gradually accepted as an alternative therapy to infused FU, similar to capecitabine (Muro et al. 2010; Yoshida et al. 2014). S-1 has the potential to improve accessibility to chemotherapy and decrease serious patient toxicities such as HFS. Placement of an ambulatory infusion pump is not absolutely necessary for patients treated with SOX plus bevacizumab. In our study, 33.3 % of the patients (n = 18) did not need an implanted port placement. Additionally, patients who received SOX plus bevacizumab returned to the hospital only once every 3 weeks. Thus, this new regimen is a promising treatment option to replace FOLFOX6 plus bevacizumab regimen, particularly for maintaining the quality of life of the patients.

In the phase I/II study of SOX regimen as the first-line treatment for metastatic CRC in Japan, Yamada et al. showed a RR of 50 % and a median PFS of 6.4 months (Yamada et al. 2008). In the phase II study of SOX regimen in Korea, Zang et al. (2009) showed a RR of 54 % and a median PFS of 8.5 months. A phase III study in South Korea showed that SOX is non-inferior to capecitabine plus oxaliplatin, with a RR of 47 % and a median PFS of 8.5 months (Hong et al. 2012). Based on the results of these previous studies, we hypothesized the expected and threshold RRs of 50 and 30 %, respectively, used to design our study.

More recently, SOFT trial demonstrated the non-inferiority of SOX plus bevacizumab to mFOLFOX6 plus bevacizumab, with a RR of 61.5 % and a median PFS of 10.2 months (Yamada et al. 2013). In the present study, the RR was 47.1 % (95 % CI 33.7–60.6 %), achieving the primary endpoint, and the median PFS was 9.2 months (95 % CI 7.6–10.8 months, Fig. 1). Recent clinical trials have reported the median OS to be more than 30 months (Yamada et al. 2013; Schwartzberg et al. 2014). In this study, the median OS was 22.5 months (95 % CI 19.4–25.9 months). Additional investigation into the OS in the future is necessary, because our median follow-up duration was only 20.2 months.

The toxicity profile of SOX plus bevacizumab is known to be different from that of mFOLFOX6 and XELOX (capecitabine/oxaliplatin). In the present study, the frequency of severe (grade 3/4) hematological toxicities including thrombocytopenia were low; however, the occurrence of anorexia was high (18.5 %). HFS was rarely observed in the present study. The occurrence of sensory neuropathy was 16.7 %, which was almost equivalent to that occurring after the administration of FOLFOX6 or XELOX. The incidence of serial complications related to bevacizumab, such as venous thrombosis, gastrointestinal perforation, and bleeding complications, was extremely low. The occurrence of hypertension was high (grade 3/4, 22.2 %); therefore, medical management was needed. Generally, adverse events were capable of being managed in the present study; however, attention to the occurrence of anorexia was necessary.

In the present study, the median RDI of oxaliplatin, S-1, and bevacizumab was 88, 84, and 90 %, respectively. In the phase I/II study of the SOX regimen in Japan, the median RDI of oxaliplatin and S-1 was 82.8 and 74.6 %, respectively (Yamada et al. 2008). In the phase II study of the SOX regimen in Korea, the median RDI of oxaliplatin and S-1 was 82 and 82 %, respectively (Zang et al. 2009). In a phase III study in South Korea, the median RDI of oxaliplatin and S-1 was 88 and 93 %, respectively (Hong et al. 2012). In SOFT trial, the median RDI of oxaliplatin, S-1, and bevacizumab was 75.5, 79.9, and 88.5 %, respectively (Yamada et al. 2013). These results suggest an almost equivalent RDI of the present study to those in previous studies.

SOFT trial already showed the non-inferiority of SOX plus bevacizumab to mFOLFOX6 plus bevacizumab in a full analysis set without patients with peritoneal disseminations (Yamada et al. 2013). The new finding of our present study was that the primary endpoint was achieved in an intention to treat set including patients with peritoneal dissemination (four patients). The efficacy and safety of the SOX plus bevacizumab regimen was also demonstrated in this prospective multicenter phase II trial conducted in 12 institutions that play a major role in regional medicine in Hiroshima, Japan.

In conclusion, the SOX plus bevacizumab regimen is effective and capable of being managed in patients with advanced/metastatic CRC in our community clinical practice, and is an option for treatment to replace the FOLFOX plus bevacizumab regimen.
